# *Methionine synthase A2756G *polymorphism may predict ulcerative colitis and *methylenetetrahydrofolate reductase C677T *pancolitis, in Central China

**DOI:** 10.1186/1471-2350-9-78

**Published:** 2008-08-13

**Authors:** Min Chen, Laurent Peyrin-Biroulet, Bing Xia, Rosa-Maria Guéant-Rodriguez, Jean-Pierre Bronowicki, Marc-André Bigard, Jean-Louis Guéant

**Affiliations:** 1 Inserm, U724, Laboratory of Cellular and Molecular Pathology in Nutrition, Faculty of Medicine, Nancy-Université, Nancy- Vandoeuvre, France; 2 Department of Gastroenterology, Zhongnan Hospital and Research Center of Digestive diseases, Wuhan University School of Medicine, Wuhan, PR China; 3 Department of Hepato-Gastroenterology, University Hospital of Nancy, Nancy- Vandoeuvre, France

## Abstract

**Background:**

The association of genetic polymorphisms related to metabolism of homocysteine with inflammatory bowel disease has been evidenced in Crohn disease and remains an open question in ulcerative colitis. We evaluated the association of the polymorphisms of MTHFR, MTR, MTRR and TCN2 genes with ulcerative colitis in Central China.

**Methods:**

168 patients were genotyped for these polymorphisms and compared to 219 matched controls.

**Results:**

*Methionine synthase *2756G allele frequency was higher in ulcerative colitis than in controls 0.15 (95% C.I. 0.11–0.19) vs 0.09 (95% C.I. 0.07 – 0.12), (P = 0.0137) and predicted ulcerative colitis risk in logistic regression, with an Odds ratio at 1.8 (95% C.I. 1.15–2.84). *Methylenetetrahydrofolate reductase* 677TT genotype was 2.7-fold more prevalent in individuals with pancolitis than in those with left colitis or proctitis, with respective percentages of 27.3 (95% C.I.16.4–42.0) and 10.5 (95% C.I. 6.3–17.1) (P = 0.0123). The carriage of 677TT or 677CT/1298AC genotypes of *methylenetetrahydrofolate reductase* was more frequent in cases with pancolitis than in subjects with left colitis or proctitis (P = 0.0048), with an Odds ratio adjusted by age and sex at 3.3 (95% C.I. 1.4–7.9), P = 0.0084) in logistic regression.

**Conclusion:**

*Methionine synthase *and *methylenetetrahydrofolate reductase* are genes of vitamin B12 and folate cellular metabolism associated respectively with risk and extent of ulcerative colitis, at least in Central China. This finding may open new insights, particularly for the potential interest in treating patients carrying the 677TT MTHFR genetic trait and a deficit in folate.

## Background

Homocysteine is a key metabolite of the one carbone metabolism, at the cross point between the remethylation pathway that produces methionine and the transulfuration pathway that produces cysteine [1]. Plasma homocysteine (t-Hcys) is influenced by genetic polymorphisms of the key enzymes of the remethylation pathway, *methylenetetrahydrofolate reductase *(*MTHFR C677T*) [2,3], *methionine synthase *(*MTR A2756G*) [4] and *methionine synthase reductase *(*MTRR A66G*) [5]. MTHFR catalyses the synthesis of methyletetrahydrofolate, the methyl donor of homocysteine and MTR and MTRR are the two key enzymes of the synthesis of methionine by remethylation of homocysteine. The *C677T *genetic polymorphism in the *MTHFR *gene is found to be associated with a thermo-labile variant enzyme that shows a reduced activity [2]. A genetic polymorphism in the *transcobalamin *gene (*TCN2 C776G*) is another genetic trait that may influence the vitamin B12 cellular delivery and consequently, homocysteine metabolism [6]. Transcobalamin is a specific plasma transporter of cobalamin (vitamin B12) and facilitates the cellular uptake of the vitamin by receptor-mediated endocytosis [6].

Hyperhomocysteinemia is recognized as a risk factor for both venous and arterial thrombosis in the general population [7,8]. In this regard, genetic determinants of homocysteine metabolism have received increasing attention over the past decade as potential contributors to the greater risk of thrombosis in inflammatory bowel disease (IBD) subjects [9]. Homocysteine has also a crucial role in cellular stress, epigenetic events, inflammatory processes and host-microbial interactions. Hyperhomocysteinemia might therefore influence the clinical history of IBD, including disease severity [9]. Mahmud and colleagues were the first to report a higher prevalence of MTHFR *677T *allele in IBD patients, compared to controls [10]. Nevertheless a number of subsequent studies have produced conflicting results, possibly related with complex interactions between environmental and genetic factors [11-17]. Ethnic and geographical variations in the distribution and phenotypic influence of *MTHFR *variants may explain, at least in part, discrepancies between case control studies on the association between *MTHFR *polymorphism and IBD [18,19]. Polymorphisms of other homocysteine metabolism-related enzymes may also contribute to the link between homocysteine metabolism and IBD patients. The influence of *MTHFR, MTR, MTRR *and *TCN2 *polymorphisms on the primary and secondary risk of ulcerative colitis have never been evaluated in a same sample population. Most of the case-control studies have evaluated Caucasian populations from North America and Europe, where the prevalence of Crohn disease is predominant, in contrast with populations from Asia, where UC is the most frequent form of IBD. In contrast to *MTHFR 677T *allele, the G allele frequency of *TCN2 *polymorphism is dramatically higher in Central China, compared with that reported in Caucasian populations [16,18,19].

The aim of this study was therefore to evaluate the influence of genetic determinants of homocysteine metabolism, *MTHFR*, *MTR*, *MTRR *and *TCN2 *with the primary risk and the clinical manifestations of UC, in a case control study of a sample population from Central China.

## Methods

### Study subjects

Eligible patients were males or females with established ulcerative colitis UC. 168 patients who were recruited from one single department (Department of Gastroenterology and Reseach center of Digestive diseases, Zhongnan Hospital of Wuhan University, Wuhan, province of Hubei, RP China) were prospectively enrolled in a case-control study, during a 2-years period. Information regarding concomitant medications was collected at study entry. Extent of UC was defined according to Montreal Classification [20]. The term "Pancolitis" means "ulcerative colitis that involves the entire colon." The UC subjects were compared to 219 healthy volunteers from a blood donor Center of Wuhan, who presented without anemia, abdominal pain, inflammation, diarrhea nor blood in the stools. The controls were extracted from a larger cohort and were matched for age and sex with UC patients. All individuals belonged to Han ethnicity and originated from the Hubei province, in Central China. This study was approved by the local ethic committee, and the subjects included gave informed consent, according to the Helsinki declaration.

### Assays and DNA genotyping procedures

Fasting venous blood was collected in EDTA-containing tubes, immediately centrifuged, and stored at -20°C until analysis. DNA was isolated from a lymphocyte-enriched fraction of whole blood with NUCLEON BACC3 for extraction of genomic DNA kit (Amersham Pharmacia Biotech, Milan, Italy). The procedures for detecting the *C677T *and *A1298C *polymorphisms of *MTHFR*, as well as the *A2756G MTR *and the *A66G MTRR *polymorphisms, were based on polymerase chain reaction (PCR) amplification, restriction cleavage and separation of the DNA fragments by 15% non denaturant polyacrylamide gel electrophoresis (SDS-PAGE), as previously described [18,19]. Genotyping of the *TCN2 C776G *polymorphism was performed by the amplification-refractory mutation system, as described recently by us [6]. DNA samples corresponding to amplified DNA of the *MTHFR*, *MTR *and *MTRR *genotypes were sequenced and subsequently used as controls in all series of genotype determination. Five % of the samples were re-genotyped to check for genotype calling consistency, with a genotyping success rate higher than 98%.

### Statistical analysis

The tested *a priori *hypotheses were the association of the genetic determinants of homocysteine metabolism with the primary risk and the clinical characteristics of the disease, respectively. Categorical variables were reported as counts, percentages and 95% confidence interval, and continuous variables as means ± SD. For categorical variables, continuity-corrected chi-square test or exact Fisher test (when variables were stratified in function of clinical characteristics) were used to assess differences. For continuous variables, a Mann-Whitney U-test was employed. The significance, odds ratios (OR) and 95% confidence interval of independent determinants regarding the risk of IBD were determined by logistic regression analysis using a model that included age, sex and the genetic and clinical variables. The residual model of logistic regression multivariate analysis considered only the variables with P-value < 0.10 in the univariate analysis. The minimal size of our sample was estimated at 150 patients, with a study power 1-β = 0.8 and α = 0.05, assuming a 1.5-fold difference in the less frequent alleles between controls and patients. P-value lower than 0.05 indicated statistical significance and Bonferroni correction for multiple testing was used as described previously, when several dependent or independent statistical tests were being performed simultaneously [18]. (Data were prospectively collected and analyzed using the Statview 5 software for Windows (SAS Institute, Berkley, California, USA) and the SPSS 10.0 software for Windows (SPSS, Paris, France). HWE was tested using the chi-square two-tailed calculation of Knud Christensen, population genetics, www.kursus.kvl.dk.

## Results

### Clinical characteristics of UC patients

There were no significant difference in sex ratio between controls and UC patients, with respectively 38% and 42% of females (P = 0.3660); the age was respectively 41 ± 15 and 41 ± 12 years (P = 0.7428). The age onset was 38 ± 15, without difference among gender. The main clinical characteristic of the UC patients are summarized in table 1. A quarter presented with an extensive form of colitis and about 57% with lesions limited to rectum and/or sigmoid. None of the cases presented with a reported thromboembolic episode.

**Table 1:** Categorical clinical characteristics of the 168 patients with ulcerative colitis.

**Characteristic**	**Number of cases**	**Percentage (95% confidence interval)**
Male sex	97	57.7 (50.2-57.7)
Past smoking	14	8.3 (5.1–13.5)
Current smoker	12	7.1 (4.2–12.1)
**Concomitant medications:**		
Steroids	50	29.8 (23.4–29.8)
Sulfasalazine	98	58.3 (50.8–65.5)
5-aminosalicylate	69	41.1 (33.9–48.6)
Antibiotics	15	8.9 (5.5–14.2)
**Disease location:**		
Rectum	78	46.4 (39.0–54.0)
Left colon	46	27.4 (21.2–34.6)
Pancolitis	44	26.2 (20.1–33.3)
**Personal history of surgery:**		
Colectomy	2	1.2 (0.4–4.2)

### Comparison of the genetic characteristics of UC patients and controls

There was no linkage desequilibrium among different genotypes. The genotype of *MTHFR C677T, A1298C*, *MTR*, *MTRR*, and *TCN *could be determined in respectively 215, 213, 218, 219, 216 of the 219 DNA samples from controls and in respectively 168, 160, 156, 164, 146 of the 168 DNA samples from UC cases. The genotype distributions of *MTHFR*, *MTR*, *MTRR*, and *TCN *polymorphisms of patients were in Hardy-Weinberg equilibrium (P-values of two tailed chi-square 0.5071, 0.8231, 0.3055, 0.7184, 0.4237, respectively) and were not influenced by gender (data not shown). *MTHFR 677T *and *1298C *alleles were in complete disequlibrium, as previously reported in other populations [3].

The genotype distributions of *MTHFR*, *MTRR *and *TCN *polymorphisms did not differ between controls and UC patients (table 2). The same observation was made for allele frequencies (table 2). In contrast, the carriers of *MTR 2756AG/GG *genotypes were more frequent in UC than in the control group, with respective percentages of 26.3 (95% C.I. 20.0–33.7) and 16.5 (12.2–22.0) (P = 0.0212). The same observation was made with the *MTR 2756G *allele frequency, with a difference even more significant (table 2). In logistic regression, carriage of *MTR 2756G *allele was an independent predictor of UC, with an Odds ratio estimated at 1.8 (table 2).

**Table 2:** Genotype frequencies and minor allele frequencies of genetic polymorphisms in ulcerative colitis patients and controls.

	**Controls** Frequencies (%)	**UC patients** Frequencies (%)	**P-value**	OR (95% CI)*
***MTHFR 677***				
***CC*^#^**	40.9	38.1		
***CT*^#^**	43.3	45.2	0.6053^a^	1.12 (0.72–1.75)
***TT***	15.8	16.7	0.6821^a^	1.13 (0.62–2.05)
**Total number of patients analysed**	215	168		
**Allele *T***	0.37	0.39	0.6027	1.09 (0.81–1.46)
***MTHFR 1298***				
***AA*^#^**	66.2	69.4		
***AC***	29.1	28.1	0.7277^a^	0.92 (0.58–1.46)
***CC***	4.7	2.5	0.2631^a^	0.51 (0.15–1.66)
**Total number of patients analysed**	213	160		
**Allele *C***	0.19	0.17	0.3455	0.86 (0.59–1.26)
***MTR 2756***				
***AA*^#^**	83.5	73.7		
***AG***	15.1	23.1	0.0422^a^	1.72 (1.02–2.92)
***GG***	1.4	3.2	0.1899^a^	2.64 (0.62–11.25)
**Total number of patients analysed**	218	156		
**Allele *G***	0.09	0.15	0.0137	1.81 (1.15–2.84)
***MTRR 66***				
***AA*^#^**	58.0	53.7		
***AG***	35.6	38.4	0.4839^a^	1.17 (0.76–1.79)
***GG***	6.4	7.9	0.4746^a^	1.34 (0.60–2.99)
**Total number of patients analysed**	219	164		
**Allele *G***	0.24	0.27	0.2401	1.18 (0.85–1.64)
***TCN2 776***				
***CC*^#^**	21.8	18.5		
***CG***	38.9	45.2	0.3358^a^	1.26 (0.79–2.01)
***GG***	39.3	36.3	0.7835^a^	1.09 (0.61–1.95)
**Total number of patients analysed**	216	146		
**Allele *G***	0.58	0.59	0.9447	1.0 (0.74–1.35)

*(OR: unadjusted odds ratio; 95 CI%: 95% confidence interval)

Abbreviations :*methylenetetrahydrofolate reductase *(*MTHFR*), *methionine synthase *(*MTR*), *methylenetetrahydrofolate reductase *(*MTRR*) and *transcobalamin *(*TCN2*)

^# ^Dominant genotype. ^a ^Comparison with dominant genotype.

### Association of genetic polymorphisms with clinical characteristics of UC patients

We next examined the association of polymorphisms of homocysteine metabolism-related enzymes with the clinical characteristics. None of the polymorphisms influenced the age onset of the disease. In univariate analysis, *MTR*, *MTRR *and *TCN *polymorphisms were not associated with location and extent of UC while *MTHFR 677TT *genotype was associated with pancolitis. The frequency of the *TT *genotype of *MTHFR C677T *polymorphism was 2.7-fold higher in UC individuals with pancolitis than in other UC cases, with respective percentages of 27.3 (95% C.I.16.4–42.0) and 10.5 (95% C.I. 6.3–17.1) (P = 0.0123). The frequency of subjects who presented with either *677TT *or the double heterozygous *677CT/1298AC *genotype was also significantly different between subjects with pancolitis and those with left colitis or proctitis, with respective percentages of 43.2 (95% C.I. 29.6–43.2) and 20.2 (95% C.I. 14.1–28.1) (P = 0.0048).

Finally, the association of gene polymorphisms of genes of the remethylation pathway with the risk of pancolitis was estimated by logistic regression analysis using a model that included age, sex, medications and genetic polymorphisms. We confirmed that the only significant independent gene predictor of extensive lesions was *MTHFR*. The sex and age adjusted odds ratio of the association between pancolitis and *MTHFR 677 TT *was estimated to 3.82 (95% C.I. 1.3–11.7, P = 0.0179). When the age and sex adjusted analysis was performed by considering the carriers of *677TT *or *C677CT/1298AC *genotypes of *MTHFR *instead of *MTHFR 677TT *only, the same conclusion was reached, with an Odds ratio at 3.3 (95% C.I. 1.4–7.9, P = 0.0084).

## Discussion

Genetic factors possibly associated with ulcerative colitis remain poorly known. This is the first study that evaluated the association of genes of the remethylation pathway of homocysteine, *MTHFR*, *MTR*, *MTRR *and *TCN2*, in a case population restricted to UC, in China. It evidenced a significant association of *MTR *and *MTHFR *variants with the primary risk and the extent of the disease, respectively; *677TT *and *C677CT*/*1298AC *genotypes of *MTHFR *were predictors of extensive colitis, while no association was found with the primary risk of UC. In contrast, *MTR 2756G *allele was associated with an increased risk of UC and had no influence on the extent of the disease. MTR and MTFHR have a complementary role in this pathway. During the remethylation of homocysteine into methionine, a methyl group provided by 5-methyltetrahydrofolate (5-methylTHF) is transferred to homocysteine by MTR. In this remethylation pathway, cobalamin (vitamin B12) is involved as an intermediate carrier of the methyl group while the 5-methylTHF is synthesized by MTHFR. The phenotypic influence of the 2756G allele on the activity of MTR has not been clearly evidenced [4]. In contrast, both *677TT *and *1298AC/677CT *genotypes are known to produce a decreased catalytic enzyme activity of MTHFR [2]. Notably, the *677TT *genotype of *MTHFR *is encoding a thermolabile variant characterized by an alanine to valine substitution at position 222, and a 50% reduction in enzyme activity [2]. As a consequence, *MTHFR C677T *polymorphism produces a decreased cellular level of methyl-THF and a cellular accumulation of homocysteine, particularly in patients with insufficient folate supply [21]. Interestingly, recent experimental findings raise the possibility that homocysteine-induced cellular and vascular stress may contribute to the maintenance of a chronic mucosal inflammatory state in IBD [9,22].

Clinical implications of the *MTHFR C677T *polymorphism include increased risk for several diseases, such as vascular, neurological and for birth defects [21,23]. Its phenotypic influence on homocysteine metabolism is neutral in subjects with sufficient folate supply, as in South Europe [18]. Similarly, the association of *MTHFR *with other diseases than IBD seems to be neutral in South European countries, where the status of populations in folate is better than in North Europe, while some studies have showed association with *MTR *and *MTRR *in these countries [23,24]. This could explain some of the discrepant results produced on association studies with IBD in populations with contrasted status in folate. Indeed, two studies performed in Northern Europe reported a significant increased frequency of the *MTHFR TT *genotype [10,25], whereas four studies in Southern Europe (two Italian, one French, one Portuguese) and one Chinese study showed no difference [11,14-17]. In contrast, we found a higher frequency of *MTRR 66A *allele in patients with Crohn's disease compared with controls, and we observed an influence of this gene variant on the extent of the disease [26]. We also evidenced that vitamin B12 was a nutritional determinant of homocysteinemia, that was under the influence of oxidative stress [26]. Consistently, a recent report showed that folate deficiency was not a predictor of homocysteine level in case of increased oxidative stress, underscoring the implication of other nutritional determinants, such as vitamin B12, under pathological conditions [27]. Taken together, these results are in agreement with our findings indicating that *methionine synthase A2756G *polymorphism, a vitamin B12-dependent enzyme, may predict ulcerative colitis. The data are also difficult to compare among contrasted populations since the frequency of these gene polymorphisms and their influence on homocysteine fluctuate greatly, worldwide [18,19]. The prevalence of the homozygous *T *allele seems to be influenced by folate status, the prevalence being the highest in Mexicans, Chinese and Italians and the lowest in West Africans [18,19]. It would be therefore useful to revisit the studies on IBD and *MTHFR *that have been published in Europe by evaluating also the association with the other gene variants related with the remethylation pathway. Finally, the discrepancies observed in previous studies on association between *MTHFR *and IBD may also correspond in part to differences in the proportion of cases with extensive colitis, as suggested by our observation that MTFHR is a predictor of pancolitis, at least in UC. This observation was in agreement with our previous study of a smaller series of UC patients [16]. In addition, the association with severity of the disease may be related with a deficit in folate and vitamin B12, as previously observed by us in Caucasians with Crohn's disease [26]. Vitamin B12 metabolism influenced Crohn's disease activity by modulating oxidative stress, measured by superoxide dismutase activity [26]. Homocysteine levels were recently correlated with activity, number of flares and duration of the disease [28]. Unfortunately, we could not determine the blood level of these determinants in the present series as no serum or plasma sample was available. Furthermore, we recently found that azathioprine therapy decreased plasma homocysteine level [26], suggesting interactions between azathioprine and homocysteine metabolisms. Genetic variation in the *MTHFR *gene may result in reduced S-adenosylmethionine concentations, leading to enhanced TPMT enzyme degradation and possibly modulating azathioprine efficacy [29].

Over the past decade, hyperhomocysteinemia and *MTHFR C677T *polymorphism have been regarded solely as potential contributors to the greater risk of thrombosis in IBD [14,15,30-32]. Our data indicated clearly that it influenced the severity of UC, independently of the risk of thrombosis, as none of the case from our series underwent a thrombosis episode.

## Conclusion

*MTR and MTHFR *are two genes of vitamin B12 and folate cellular metabolism associated respectively with the primary risk and the severity of UC, at least in central China. These findings might open new insights into the pathogenesis and assessment of UC, particularly for the potential interest in treating the patients presenting with the *677TT MTHFR *genetic trait and a deficit in folate. However, our results await confirmation in a large cohort of patients with UC.

## Competing interests

The authors declare that they have no competing interests.

## Authors' contributions

J–LG was the guarantor and supervisor of the study. MC, LP–B, BX and J–LG contributed to conception and design, recruitment of patients, analysis and interpretation of data and drafting of the manuscript. R–MG–R carried out the biological and genetics analyses, interpretation of data, statistical analyses, revising of the manuscript. BX and MC recruited the patients and M–AB and J–PB contributed in interpretating the data. LP–B and J–LG revised the manuscript. All authors read and approved the final manuscript.

## Pre-publication history

The pre-publication history for this paper can be accessed here:


